# Romantic relationship obsessive-compulsive doubts, perfectionism, and DSM-5 personality traits in LGB people: a comparison with heterosexual individuals

**DOI:** 10.3389/fpsyg.2024.1187179

**Published:** 2024-02-21

**Authors:** Nicole Loren Angelo, Greta Brullo, Alessandro Marsiglia, Alessandra Tirelli, Elisa Piroddi, Chiara Viti, Ilaria Aicardi, Andrea Pozza

**Affiliations:** ^1^Department of Medical Sciences, Surgery and Neurosciences, University of Siena, Siena, Italy; ^2^Miller Institute of Cognitive Behavioral Therapy, Genoa, Italy; ^3^RSA “Casa S.s. Concezione”, Genoa, Italy; ^4^Psychology Unit, Azienda Ospedaliera Universitaria Senese AOUS, Siena, Italy

**Keywords:** relationship obsessive-compulsive disorder, LGB, intimate relationships, personality, perfectionism, partner-focused obsessions, relationship-oriented obsessions

## Abstract

**Introduction:**

Relationship Obsessive-Compulsive Disorder is characterized by the presence of relationship-centered or partner-focused obsessions and compulsions that determine a great sense of doubt toward the partner or the relationship. Personality characteristics, including perfectionism, are involved in the development of Relationship Obsessive-Compulsive Disorder, and could predispose the individual to excessive doubts and preoccupations regarding the “adequacy of the couple” or the physical appearance of one’s partner. Evidence from epidemiological research shows that the LGB community can present a high risk and prevalence of obsessive-compulsive symptoms and recent research demonstrated the usefulness of the DSM-5 personality model in understanding the personality of sexual minorities. However, further research is necessary to deepen our knowledge of the relationship between these variables in the LGB community. The aim of the present study was to compare a group of heterosexual individuals to a group of LGB individuals regarding personality traits, perfectionism, and relationship obsessive-compulsive symptoms.

**Methods:**

A total of 200 participants, 98 in the heterosexual group and 102 in the LGB group, were enrolled in the study and completed a psychological battery comprised of the Obsessive-Compulsive Inventory-Revised, Personality Inventory for DSM-5, Relationship Obsessive-Compulsive Inventory, Partner-Related Obsessive-Compulsive Symptom Inventory, and Multidimensional Perfectionism Scale.

**Results:**

The results show that LGB individuals tend to report greater feelings of doubt regarding the partner’s love, more negative emotions (Negative Affect) and Antagonism, and greater perfectionism traits compared to heterosexual individuals.

**Conclusion:**

These findings underline the necessity to consider the implementation of personalized interventions in clinical practice and the importance of initiating early preventive programs in sexual minority communities.

## 1 Introduction

Relationship Obsessive-Compulsive Disorder (ROCD) is characterized by the presence of relationship-centered or partner-focused obsessions and compulsions. Obsessions are recurrent and persistent thoughts, impulses, or images, experienced as intrusive or undesired, that cause a prominent sense of discomfort. The individual, therefore, attempts to ignore or repress such thoughts, impulses or images or neutralize them with mental or externalized behaviors – compulsions that require a notable expenditure of time, interfering with normal functioning and cause clinically significant distress and/or compromise functioning in important areas of life (American Psychiatric Association, 2013; Coluccia et al., 2015).

In ROCD the individual feels a great sense of doubt toward the partner or the relationship itself. The obsessions and compulsions manifest themselves in thoughts, images or urges, and are accompanied by a repetitive checking of one’s feelings and/or thoughts toward the partner or the relationship itself, constant reassurance seeking among other typical behaviors (Doron et al., 2014). Persistent doubts about the relationship or the partner could influence the quality and satisfaction of the relationship and the interpersonal dynamics between the couple (Doron et al., 2012a,b,c, 2014; Kabiri et al., 2016). Independently of the presence of an OCD diagnosis, relationship-related obsessive-compulsive symptoms determine distress, depression, negative affect, and alter functioning in intimate relationships (Doron et al., 2012a,b,c).

Doron et al. (2014) have identified two different symptomatologic forms of ROCD. Relationship-centered obsessive-compulsive traits are characterized by doubt and preoccupations (i.e., “Is this the right relationship?”; Doron et al., 2014) that could be relative to the feelings that the person has toward the partner, the feelings the partner has toward the person, and/or the evaluation of the relationship in terms of right or wrong (Doron et al., 2012a). Partner-focused obsessive-compulsive traits consist of obsessive doubts and preoccupations regarding the perceived flaws in the partner that could refer to different dimensions: physical appearance, intellectual skills, social and or personality characteristics (i.e., “Is she beautiful enough?”; Doron et al., 2014). Both forms are not necessarily mutually exclusive (Doron et al., 2012b); they could both be present simultaneously and maintain themselves through time or they could be individually present, and the individual could pass from one form to the other (Doron et al., 2014).

### 1.1 Personality characteristics and relationship obsessive-compulsive disorder

The DSM-5 Personality and Personality Disorders workgroup developed a hybrid dimensional model of maladaptive personality traits, which derives a categorial diagnosis from dimensional traits. The model comprises five higher-order domains of pathological personality traits (Table 1; American Psychiatric Association, 2013) and two areas of impairment in personality functioning (Self and Interpersonal) from which it is possible to derive four specific areas: identity, self-direction, empathy, and intimacy (Skodol et al., 2015). In this dimensional model a personality disorder is defined by the combination of clinically significant impairments in functioning and one or more pathological trait (Kreuger and Hobbs, 2020). For example, Avoidant Personality Disorder is diagnosed when there are low levels of self-esteem (identity), unrealistic standards (self-direction), preoccupation with criticism (Empathy), reluctance to get involved with others (intimacy), and a combination of Negative Affectivity and Detachment (Skodol et al., 2015). From this dimensional model it is possible to detect one of the seven possible personality disorders (Quilty et al., 2013). A key advantage of this new model is that personality disorders can be conceptualized in terms of specific constellations of maladaptive traits, rather than being distinct constructs from each other and from normal personality (Hopwood et al., 2013). From this hybrid model an instrument for the assessment of personality traits was created: the Personality Inventory for the DSM-5 (PID-5; Krueger et al., 2011). Given the PID-5’s ability in capturing abnormal (Krueger and Markon, 2014) and extreme (Krueger et al., 2011) ranges of personality dimensions, and its empirical nature (Harkness et al., 2012) this measure is a valuable clinical tool and has, therefore, been used in our study.

**Table 1:** The 5 personality domains identified by the DSM-5 personality and personality disorders workgroup (American Psychiatric Association, 2013).

***Negative affect – emotional stability***	Frequent and intense experiences of high levels of negative emotions (i.e., anxiety, depression, guilt/embarrassment, worry, anger) and relative behavioral (i.e., self-harm) and inter-personal (i.e., dependent behavior) manifestations.
***Detachment – extroversion***	Avoidance of socio-emotional experiences expressed as retiring from interpersonal interactions (from daily casual interactions, to friendships, and intimate relationships) and impaired capability of feeling and expressing emotions.
***Antagonism – availability***	Behaviors that put the individual at odds with other people like an exaggerated sense of self-importance and the expectation to deserve special treatment from others.
***Disinhibition – conscientiousness***	Orientation toward immediate gratification and impulsive behaviors without considering passed experiences or future consequences.
***Psychoticism – mental lucidity***	Manifestation of a wide range of culturally incongruent, odd, eccentric, or unusual behaviors and thoughts.

Personality characteristics are influenced by the environment and the quality of the primary relationship established between parent and child. The attachment style developed during infancy is a similar construct to personality. Insecure attachment behaviors (anxious, avoidant, or disorganized attachment styles – see Ainsworth and Witting, 1969; Ainsworth et al., 1978; Main and Solomon, 1986) could exacerbate doubts and concerns regarding the relationship (Mikulincer and Shaver, 2007). Insecure attachment is characterized by anxiety-related behaviors, consisting of a hyper-activation of the attachment system (i.e., constant attempts to secure care, love, and support from the partner); or avoidant behaviors consisting of a de-activation of the attachment system (i.e., denying or suppressing thoughts and emotions related to attachment; Mikulincer and Shaver, 2003).

Insecure attachment behaviors are linked to dysfunctional cognitive processes related to OCD (Obsessive Compulsive Cognitions Working Group, 1997; Pozza et al., 2013, 2021). In fact, Doron et al. (2009) propose that insecure attachment styles are associated with emotion dysregulation in situations that challenge sensitive self-domains. For example, perfectionist tendencies could lead to preoccupations in terms of the “rightness” of the relationship; intolerance of uncertainty could lead to question one’s feelings and emotions toward the partner (Lazarov et al., 2010); the individuals’ interpretations of the partners’ feelings toward them could be biased in terms of overestimation of threat, leading to doubts concerning the partners’ feelings. As a consequence of such biases, the individual could repeatedly seek reassurance from the partner or others, check specific relationship-oriented behaviors, or avoid situations that could determine doubt, causing a significant strain on the relationship (Doron et al., 2012a, 2013). In particular, personality traits characterized by perfectionism, strongly associated with general obsessive-compulsive symptoms (Obsessive Compulsive Cognitions Working Group, 2001, 2005; Pozza et al., 2019; Miegel et al., 2020; Lu, 2022), could represent a relevant predisposing factor for the development of excessive doubts and preoccupations toward one’s partner and/or relationship regarding the “adequacy of the couple” or the physical characteristics or the personality of one’s partner (Lazarov et al., 2010).

Theoretical models and empirical data by factor analyses indicate that perfectionism is a multidimensional construct including Perfectionistic Strivings and Evaluative Concerns (Frost et al., 1990; Flett and Hewitt, 2002). Perfectionistic strivings refer to those facets of perfectionism that relate to perfectionistic personal standards and a self-oriented striving for perfection. They include self-oriented perfectionism (i.e., demanding perfection of oneself) and personal standards (i.e., setting unreasonably high personal standards and goals) (Frost et al., 1990; Hewitt and Flett, 1991). This dimension was found to be related to both negative and positive processes (i.e., adaptive coping) and outcomes (i.e., psychological adjustment; Stoeber and Otto, 2006). Conversely, perfectionistic concerns were found to be related to negative outcomes as well as socially prescribed perfectionism (i.e., perceiving others as demanding perfection of oneself), concern over mistakes (i.e., adverse reactions to failures), doubts about actions (i.e., doubts about performance abilities), and self-criticism (the tendency to assume blame and feel self-critical toward the self; Frost et al., 1990; Hewitt and Flett, 1991).

### 1.2 Mental health and personality in the LGB community

A large amount of long-standing data shows that sexual minorities, such as the LGB community, can face increased psychological problems compared with the heterosexual population (Balsam et al., 2005; Chakraborty et al., 2011; Lucassen et al., 2017; Parker and Harriger, 2020; Travers et al., 2020; Bhugra et al., 2022).

Evidence from epidemiological research shows that sexual minorities such as the LGB community can present a high risk and prevalence of psychopathological symptoms and traits, with studies suggesting that the prevalence of obsessive-compulsive symptoms in LGB people can be higher than in the heterosexual community (Pinciotti and Orcutt, 2021) and range from 2.6% up to 14% (Chakraborty et al., 2011; Ciocca et al., 2018). However, more data are needed.

Some data suggests that mental health problems in LGB people can be linked to personality traits to some extent (Cramer et al., 2016). Recent research findings in the field demonstrated the usefulness of DSM-5 personality model in understanding personality of sexual minorities including LGB populations. In a very large sample of women and men, Russell et al. (2017) found that lesbians and bisexual women and men had greater scores on several PID-5 domains relative to heterosexual peers. Lesbians and bisexual women had significantly greater scores than heterosexual peers on every domain except antagonism. Additionally, bisexual men had significantly greater scores on psychoticism than heterosexual men, and on detachment than heterosexual and gay men.

Regarding perfectionism, Meyer’s minority stress model (2003) suggests that individuals belonging to a sexual minority undergo a number of stressors that could lead to a worry about making mistakes and, therefore, bring to overcompensation in achievement-related domains [see the “Best Little Boy in the World hypothesis of Tobias (1976)], consequently developing maladaptive perfectionistic strategies (Ying et al., 2022).

### 1.3 Rationale

The available literature underlines a greater vulnerability of the LGB community toward obsessive-compulsive symptoms and the presence of dysfunctional personality traits, as compared to individuals with a heterosexual orientation. Indeed, several studies (Meads et al., 2009; Chakraborty et al., 2011; Przedworski et al., 2015; Ciocca et al., 2018), report a greater prevalence of obsessive-compulsive symptoms in LGB individuals. Non-heterosexual individuals also seem to report a greater prevalence of dysfunctional personality traits (Wang et al., 2014; Cramer et al., 2016; Russell et al., 2017) and greater maladaptive perfectionism (Ying et al., 2022). However, further research is necessary to investigate the interrelations between sexual orientation and romantic relationship obsessive-compulsive symptoms.

### 1.4 Aims and hypotheses

The objective of the present study was (i) to compare a group of participants with a heterosexual orientation to a group of participants with an LGB orientation regarding different relationship obsessive-compulsive, perfectionism, and personality traits; (ii) explore the role of perfectionism and personality domains in the presence of relationship obsessive-compulsive traits in both groups.

Based on the previously mentioned literature that underlines a greater vulnerability of LGB individuals toward the development of obsessive-compulsive symptoms, we hypothesize that the LGB group would present greater relationship obsessive-compulsive traits and obsessive-compulsive symptoms as compared to the heterosexual group. Also, the available literature highlights a greater prevalence of dysfunctional personality traits, as measured by the PID-5, in LGB individuals compared to individuals with a heterosexual orientation. Therefore, in line with the findings of Russell et al. (2017) we hypothesize that the LGB group would refer higher levels of Negative Affect, Detachment, Disinhibition, and Psychoticism. Further, we hypothesize that the LGB group would present greater perfectionistic traits compared to the heterosexual one. Finally, we hypothesize that such perfectionistic traits would correlate with relationship-oriented and partner-focused obsessive-compulsive traits, as measured by the ROCI and the PROCSI, respectively.

## 2 Methods

### 2.1 Eligibility criteria and procedure

The following eligibility criteria were applied to include participants in the present study: (i) participants had to be 18 years of age or older, (ii) be engaged in an affective relationship for at least 6 months. Potential participants were recruited through public services, such as sport centers or entertainment centers.

Based on these inclusion criteria, a total of 200 participants (*n* = 98 in the heterosexual group; *n* = 102 in the LGB group), equally distributed by gender, took part in the study.

### 2.2 Measures

A psychological battery of self-report questionnaires was administered in-person to the participants. The battery contained measures evaluating OCD symptoms, personality traits, relationship-centered and partner-focused obsessive-compulsive symptoms, and perfectionism: Obsessive-Compulsive Inventory-Revised (OCI-R; Foa et al., 2002); Personality Inventory for the DSM-5 (PID-5; Fossati et al., 2017); Relationship Obsessive-Compulsive Inventory (ROCI; Doron et al., 2012a); Partner-Related Obsessive Compulsive Symptom Inventory (PROCSI; Doron et al., 2012a); Multidimensional Perfectionism Scale (MPS; Hewitt and Flett, 1996).

#### 2.2.1 Personality inventory for the DSM-5 – adult

The Italian version of the PID-5 was used (Fossati et al., 2017). The PID-5 comprises 202 items that evaluates personality using 5 domains: Negative Affect (Example item: “My emotions sometimes change for no good reason”), Detachment (Example item: “I prefer not to get too close to people”), Antagonism (Example item: “I’m good at making people do what I want them to do”), Disinhibition (Example item: “Other see me as irresponsible”), and Psychoticism (Example item: “I sometimes have heard things that others could not hear”). The PID-5 scoring guide-lines report that higher scores represent a greater dysfunction or compromise in that specific trait (Fossati et al., 2015).

#### 2.2.2 Obsessive-compulsive inventory-revised

The OCI-R (Foa et al., 2002) is a self-report questionnaire that quantifies the six fundamental dimensions that characterize Obsessive-Compulsive Disorder: Washing (Example item: “I sometimes have to was or clean myself simply because I feel contaminated”), Obsessing (Example item: “I find it difficult to control my own thoughts”), Hoarding (Example item: “I collect things I do not need”), Ordering (Example item: “I need things to be arranged in a particular way”), Checking (Example item: “I repeatedly check doors, windows, drawers, etc.”), and Mental Neutralizing (Example item: “I feel compelled to count while I am doing things”). The Italian version of the OCI-R (Sica et al., 2009) presents acceptable internal consistency (α > 0.70) and good test–retest reliability (*r* > 0.70).

#### 2.2.3 Relationship obsessive-compulsive inventory

The ROCI is a self-report instrument that evaluates the presence of obsessive-compulsive symptoms in the romantic relationship (Doron et al., 2012a) using 3 subscales: Love for Partner (feelings toward current partner, Example item: “I keep thinking if I truly love my partner”), Adequacy of the Relationship (thoughts regarding the appropriateness of the relationship, Example item: “I ask myself if this relationship is the right one”), Love of Partner (feelings the partner has toward the individual, Example item: “I doubt my partner’s love for me”). The ROCI was developed on the hypothesis that obsessive-compulsive phenomena influence intimate relationships when the central theme of the symptoms is the relationship. The Italian version of the ROCI (Melli et al., 2018b) shows very good internal consistency (α > 0.81 for all subscales), good construct and criterion validity and excellent diagnostic sensitivity (Melli et al., 2018a).

#### 2.2.4 Partner-related obsessive-compulsive symptom inventory

The partner-related obsessive-compulsive symptom inventory (PROCSI) (Doron et al., 2012a), is a self-report instrument that measures obsessions and neutralizing behaviors regarding partner’s flaws. The PROCSI takes into consideration six domains: Physical Appearance (Example item: “When I am with my partner I find it hard to ignore her physical flaws”), Social Abilities (Example item: “I repeatedly evaluate my partner’s social functioning”), Morality (Example item: “I keep looking for evidence that my partner is moral enough”), Emotional Stability (Example item: “I am bothered by doubts about my partner’s emotional stability”), Intelligence (Example item: “The thought that my partner is not intelligent enough bothers me greatly”), and Competence (Example item: “I keep looking for evidence of my partner’s occupational success”).

The Italian version of the PROCSI was developed by Melli et al. (2018b) and shows good internal consistency (α > 0.77 for all subscales), good construct and criterion validity, and excellent diagnostic sensitivity (Melli et al., 2018a).

#### 2.2.5 Multidimensional perfectionism scale

The MPS (Hewitt et al., 1991), is a self-report measure used to evaluate four dimensions of perfectionism: Concern over Mistakes and Doubts about Actions (i.e., negative reactions to mistakes, perception of even minor errors as failure, and repeatedly doubting the quality of one’s performance; example item: “I should be upset if I make a mistake”), concerns with “precision, order and organization” (i.e., usually not referring to pathological functioning, the tendency to organize behavior and be neat; example item: “Organization is very important to me”), Excessively High Personal Standards (i.e., the tendency to set excessively high standards; example item: “If I do not set the highest standards for myself, I am likely to end up a second-rate person.”), Parents’ Expectations and Evaluation (i.e., perceiving one’s parents as having high expectations or being excessively critical; example item: “As a child, I was punished for doing things less than perfectly”). The Italian version of the MPS (Lombardo, 2008) shows good internal consistency (α > 0.75), good concurrent validity, and good construct validity.

### 2.3 Data analyses

A series of independent-group Student *t-*tests were calculated on the scores of the PID-5, PROCSI, ROCI, OCI-R, and MPS to compare the heterosexual group to the LGB group.

The associations between the scores of the PID-5, PROCSI, ROCI, OCI-R, and MPS were calculated using Pearson’s bivariate correlation. Correlation coefficients were calculated, separately in the heterosexual and the LGB group. Values on the correlation coefficients were interpreted according to the following criteria provided by Cohen (1988): 0 < |*r*| < 0.30 = weak; 0.30 < |*r*| < 0.50 = moderate; 0.50 < |*r*| < 0.70 = strong; 0.70 < |*r*| < 1 = very strong.

A multiple linear regression analysis with stepwise input was carried out separately in the two groups to identify the possible predictors of relationship-centered and partner-focused obsessive-compulsive symptoms in both groups.

The statistical analyses were conducted using SPSS software.

## 3 Results

### 3.1 Descriptive characteristics

A total of 200 participants enrolled from the general population took part in the study. The sample was equally distributed for gender (*n*_males_ = 100; *n*_females_ = 100) and domestic partnership and presented an age range between 18 and 76 (*M* = 32.11, *SD* = 10.77). Other descriptive characteristics of the sample are presented in Table 2.

**Table 2:** Socio-demographic characteristics of the total sample (*n* = 200).

	*n* (%)	*M*	*SD* (Range)
**Age**	18–76	32.11	10.77 (18–76)
**Gender**			
Male	100 (50)		
Female	100 (50)		
**Sexual orientation**			
Heterosexual	98 (49)		
LGB	102 (51)		
**Degree**			
Middle-school	19 (9.5)		
Highschool	108 (54)		
Graduate degree	73 (36.5)		
**Occupation**			
University student	80 (40)		
Unemployed	19 (9.5)		
Retired	3 (1.5)		
Other	1 (0.5)		

LGB, lesbian – gay – bisexual.

### 3.2 Group differences on the outcome measures

The *t* Student test conducted on the points of the ROCI underlined statistically significant differences regarding sexual orientation, specifically, on the scores on the ROCI – Love for Partner [*t*_(198)_ = −1.99; *p* < 0.05; Table 3].

**Table 3:** Group differences on the outcome measures (*n* = 200).

	**Heterosexual** **(*n* = 98)**			**LGB** **(*n* = 102)**			***t***	**gdl**	***p***
	***n***	***M***	**ds**	***n***	**M**	**ds**			
**PID-5 negative affect**	**98**	**1.03**	**0.51**	**102**	**1.34**	**0.54**	**−4.14**	**198**	**<0.001**
PID-5 detachment	98	0.84	0.56	102	0.92	0.55	−1.03	198	0.31
**PID-5 antagonism**	**98**	**0.89**	**0.52**	**102**	**1.07**	**0.61**	**−2.25**	**198**	**<0.05**
PID-5 disinhibition	98	0.91	0.51	102	0.93	0.40	−0.31	198	0.76
PID-5 psychoticism	98	0.58	0.44	102	0.65	0.33	−1.44	198	0.25
PROCSI morality	98	2.73	2.92	102	2.27	2.29	1.24	198	0.22
PROCSI social abilities	98	3.49	2.88	102	2.99	2.45	1.32	198	0.19
PROCSI emotional stability	98	2.86	2.77	102	2.84	2.40	0.04	198	0.97
PROCSI competence	98	2.68	3.03	102	2.85	2.57	−0.43	198	0.67
PROCSI physical appearance	98	2.82	3.10	102	2.47	2.38	0.89	198	0.38
PROCSI intelligence	98	3.22	2.92	102	2.84	2.45	1.00	198	0.32
PROCSI total	98	20.11	14.90	102	18.08	11.73	1.08	198	0.28
ROCI love for partner	98	3.84	3.79	102	4.08	3.70	−0.46	198	0.65
ROCI adequacy of the relationship	98	4.22	3.63	102	4.50	3.90	−0.52	198	0.61
**ROCI love of partner**	**98**	**3.62**	**3.11**	**102**	**4.54**	**3.39**	**−1.99**	**198**	**<0.05**
ROCI total	98	11.68	9.52	102	13.12	10.19	−1.03	198	0.31
**OCI-R hoarding**	**98**	**2.00**	**2.34**	**102**	**2.62**	**2.12**	**−2.81**	**198**	**<0.01**
OCI-R Checking	98	1.81	2.23	102	2.84	2.58	−1.96	198	0.05
**OCI-R ordering**	**98**	**2.09**	**2.33**	**102**	**2.97**	**2.71**	**−3.04**	**198**	**<0.01**
**OCI-R mental neutralizing**	**98**	**1.17**	**1.67**	**102**	**1.64**	**1.88**	**−2.45**	**198**	**<0.05**
OCI-R washing	98	1.38	1.74	102	1.72	1.87	−1.85	198	0.07
OCI-R obsessing	98	2.32	2.74	102	2.84	2.44	−1.32	198	0.17
OCI-R total	98	10.77	9.77	102	14.63	9.66	−1.44	198	0.15
**MPS concern over mistakes and doubts about actions**	**98**	**34.30**	**7.66**	**102**	**36.84**	**7.82**	**−2.33**	**198**	**<0.05**
**MPS organizations**	**98**	**17.10**	**4.59**	**102**	**18.85**	**3.92**	**−2.90**	**198**	**<0.01**
MPS Personal Standards	98	21.80	4.88	102	20.64	3.75	1.89	198	0.06
**MPS parental expectations and criticism**	**98**	**20.40**	**5.76**	**102**	**22.75**	**5.45**	**−2.96**	**198**	**<0.01**

PID-5, Personality Inventory for DSM-5; PROCSI, Partner-Related Obsessive–Compulsive Symptom Inventory; ROCI, Relationship Obsessive-Compulsive Inventory; OCI-R, Obsessive Compulsive Inventory – Revised; MPS, Multidimensional Perfectionism Scale. Bold values mean statistically significant values.

Statistically significant differences emerged between the two groups on OCI-R Hoarding [*t*_(198)_ = −2.81; *p* < 0.01], Ordering [*t*_(198)_ = −3.04; *p* < 0.01], and Mental Neutralizing [*t*_(198)_ = −2.45; *p* < 0.05; Table 3].

Statistically significant differences emerged for MPS – Preoccupation for error and doubts about actions [*t*_(198)_ = −2.33; *p* < 0.05], MPS – Organizations [*t*_(198)_ = −2.90; *p* < 0.01], and MPS - Parental expectations and criticism [*t*_(198)_ = −2.96; *p* < 0.01; Table 3].

Statistically significant differences emerged for PID-5 Negative Affect [*t*_(198)_ = −4.14; *p* < 0.001] and PID-5 Antagonism [*t*_(198)_ = −2.25; *p* < 0.05; Table 3].

No statistically significant group differences emerged for the PROCSI scores.

### 3.3 Correlations between the outcome measures in the heterosexual group

The scores obtained on the PROCSI show statistically significant positive correlations with all the values of the PID-5 (Table 4). Specifically, strong positive correlations emerged for PID-5 Antagonism and PROCSI Morality (*r* = 0.51, *p* < 0.001), PROCSI Emotional Stability (*r* = 0.58, *p* < 0.001), PROCSI Physical Appearance (*r* = 0.60, *p* < 0.001), and PROCSI Total (*r* = 0.56, *p* < 0.001). In Table 4 the other weak and moderate correlations between the PID-5 and PROCSI scores are presented.

**Table 4:** Correlations between the PID-5, PROCSI, ROCI, OCI-R, and MPS in the heterosexual group (*n* = 98).

	ROCI love for partner	ROCI adequacy of the relationship	ROCI love of partner	ROCI total	PROCSI morality	PROCSI social abilities	PROCSI emotional stability	PROCSI competence	PROCSI physical appearance	PROCSI intelligence	PROCSI total
OCI-R hoarding	0.23*	0.17	0.14	0.20*	0.15	0.21*	0.15	0.12	0.08	0.26**	0.18
OCI-R checking	0.27**	0.17	0.24*	0.25*	0.25*	0.17	0.21*	0.19	0.18	0.29**	0.25*
OCI-R ordering	0.22*	0.19	0.27**	0.25*	0.13	0.28**	0.12	0.27**	0.15	0.30**	0.24*
OCI-R mental neutralizing	0.12	0.01	0.02	0.06	0.20*	0.18	0.19	0.26*	0.12	0.17	0.22*
OCI-R washing	0.23*	0.12	0.16	0.19	0.24*	0.14	0.19	0.21*	0.22*	0.24^*^	0.25*
OCI-R obsessing	0.42***	0.35**	0.28**	0.39***	0.17	0.19	0.28**	0.18	0.18	0.28^**^	0.24*
OCI-R total	0.35***	0.24*	0.26**	0.32**	0.25*	0.26**	0.26*	0.27**	0.20	0.35***	0.31**
MPS concern over mistakes and doubts about actions	0.31**	0.33**	0.40***	0.38***	0.31**	0.36***	0.32**	0.44***	0.39***	0.37***	0.42***
MPS organizations	0.09	0.05	0.16	0.11	−0.03	0.27**	0.07	0.14	−0.04	0.13	0.12
MPS personal standards	−0.19	−0.16	−0.21*	−0.20*	−0.11	0.29**	−0.15	−0.18	−0.12	−0.19	−0.21*
MPS parental expectations and criticism	0.27**	0.29**	0.27**	0.31**	0.34**	0.20*	0.28**	0.26*	0.30**	0.34**	0.32**
PID-5 negative affect	0.33**	0.34**	0.31**	0.37***	0.24*	0.17	0.21*	0.21*	0.09	0.27**	0.23*
PID-5 detachment	0.26*	0.31**	0.29**	0.31**	0.32**	0.28**	0.22*	0.31**	0.24*	0.42***	0.35***
PID-5 antagonism	0.57***	0.45***	0.38***	0.52***	0.51***	0.35***	0.58***	0.45***	0.60***	0.48***	0.56***
PID-5 disinhibition	0.45***	0.46***	0.26*	0.44***	0.40***	0.28**	0.46***	0.25*	0.44***	0.31**	0.42***
PID-6 psychoticism	0.34**	0.37***	0.17	0.33**	0.34**	0.41***	0.34**	0.30**	0.21*	0.46***	0.40***

PID-5, personality inventory for DSM-5; PROCSI, partner-related obsessive-compulsive symptom inventory; ROCI, relationship obsessive-compulsive inventory; OCI-R, obsessive compulsive inventory – revised; MPS, multidimensional perfectionism scale.*p < 0.05; **p < 0.01; ***p < 0.001.

Significant positive correlations emerged between the scores on the PROCSI and the OCI-R (Table 4). More specifically, the most statistically significant positive moderate correlation emerged between PROCSI Intelligence and OCI-R total (*r* = 0.35, *p* < 0.001; See Table 4 for more details).

Statistically significant correlations emerged between the scores on the MPS and the PROCSI (Table 4). In particular, statistically significant positive moderate correlations were found MPS Concerns over mistakes and doubts about actions and PROCSI Social Abilities (*r* = 0.36, *p* < 0.001), PROCSI Competence (*r* = 0.44, *p* < 0.001), PROCSI Physical Appearance (*r* = 0.39, *p* < 0.001), PROCSI Intelligence (*r* = 0.37, *p* < 0.001), and PROCSI Total (*r* = 0.42, *p* < 0.001; See Table 4 for more detailed results).

Statistically significant strong positive correlations emerged between the PID-5 Antagonism scores and ROCI Love for Partner (*r* = 0.57, *p* < 0.001) and ROCI Total (*r* = 0.52, *p* < 0.001). Statistically significant moderate positive correlations emerged between the PID-5 Antagonism and ROCI Adequacy of the relationship (*r* = 0.45, *p* < 0.001) and ROCI Partner’s Love (*r* = 0.38, *p* < 0.001). Positive statistically significant moderate correlations emerged between the PID-5 Disinhibition and ROCI Love for Partner (*r* = 0.45, *p* < 0.001), ROCI Adequacy of the relationship (*r* = 0.46, *p* < 0.001), and ROCI Total (*r* = 0.44, *p* < 0.001). PID-5 Negative Affect is positively, significantly, and moderately correlated with ROCI Total (*r* = 0.37, *p* < 0.001) and PID-5 Psychoticism is positively, significantly, and moderately correlated with ROCI Adequacy of the relationship (*r* = 0.37, *p* < 0.001). See Table 4 for more detailed results.

The scores obtained on the OCI-R show statistically significant moderate correlations with the scores of the ROCI. Specifically, OCI-R Obsessing and OCI-R Total are positively and significantly correlated with ROCI Love for Partner (*r* = 0.42, *p* < 0.001; *r* = 0.35, *p* < 0.001), respectively. A moderate statistically significant positive correlation also emerged for OCI-R Obsessing and ROCI Total (*r* = 0.39, *p* < 0.001; Table 4).

Significant positive correlations emerged between the scores on the ROCI and the MPS (Table 4). More specifically, moderate statistically significant positive correlations emerged between MPS Preoccupation for error and doubts about actions (*r* = 0.40, *p* < 0.001) and ROCI Total (*r* = 0.38, *p* < 0.001; See Table 4 for more details).

### 3.4 Correlations between the outcome measures in the LGB group

The scores on the PID-5 Antagonism presented moderate statistically significant positive correlations with PROCSI Morality (*r* = 0.47, *p* < 0.001), PROCSI Emotional Stability (*r* = 0.45, *p* < 0.001), PROCSI Competence (*r* = 0.40, *p* < 0.001) and PROCSI Total (*r* = 0.39, *p* < 0.001; see Table 5 for more detailed results).

**Table 5:** Correlations between the PID-5, PROCSI, ROCI, OCI-R, and MPS in the LGB group (*n* = 102).

	ROCI love for partner	ROCI adequacy of the relationship	ROCI love of partner	ROCI total	PROCSI morality	PROCSI social abilities	PROCSI emotional stability	PROCSI competence	PROCSI physical appearance	PROCSI intelligence	PROCSI total
OCI-R hoarding	0.25*	0.30**	0.14	0.25*	0.13	0.31**	0.24*	0.18	0.17	0.26**	0.27**
OCI-R checking	0.23*	0.26*	0.15	0.23*	0.12	0.18	0.24*	0.16	0.23*	0.26*	0.23*
OCI-R ordering	0.10	0.08	0.03	0.08	0.21*	0.35***	0.22*	0.19	0.26**	0.25*	0.30**
OCI-R mental neutralizing	0.01	−0.03	−0.05	−0.03	0.04	−0.07	−0.02	0.04	0.13	−0.07	0.00
OCI-R washing	0.10	0.04	0.07	0.07	0.28**	0.16	0.20*	0.24*	0.32**	0.27*^*^	0.28**
OCI-R obsessing	0.46***	0.50***	0.39***	0.49***	0.05	0.32**	0.29**	0.07	0.24*	0.33^**^	0.26**
OCI-R total	0.28**	0.28**	0.18	0.27**	0.19	0.31**	0.29**	0.21*	0.32**	0.32**	0.33**
MPS concern over mistakes and doubts about actions	0.13	0.14	0.09	0.13	0.22*	0.15	0.35***	0.17	0.30**	0.23*	0.25*
MPS organizations	0.12	0.11	0.06	0.11	0.18	0.23*	0.22*	0.20*	0.17	0.21*	0.24*
MPS personal standards	−0.12	0.23*	−0.15	−0.17	−0.33**	−0.27**	−0.24*	−0.11	−0.30**	−0.23*	−0.26**
MPS parental expectations and criticism	0.07	−0.01	−0.08	0.05	0.30**	0.24*	0.29**	0.23*	0.30**	0.18	0.28**
PID-5 negative affect	0.42***	0.48***	0.48***	0.50***	0.20*	0.32**	0.33**	0.18	0.36***	0.30**	
PID-5 detachment	0.54***	0.51***	0.55***	0.58***	0.26**	0.35***	0.21*	0.12	0.39***	0.39**	
PID-5 antagonism	0.18	0.10	0.17	0.16	0.47***	0.32**	0.45***	0.40***	0.31**	0.39***	
PID-5 disinhibition	0.35***	0.38***	0.43***	0.42***	0.15	0.23*	0.25*	0.01	0.06	0.16	
PID-6 psychoticism	0.36***	0.31**	0.27**	0.34**	0.11	0.32**	0.26**	−0.02	0.16	0.23*	

PID-5, personality inventory for DSM-5; PROCSI, partner-related obsessive-compulsive symptom inventory; ROCI, relationship obsessive-compulsive inventory; OCI-R, obsessive compulsive inventory – revised; MPS, multidimensional perfectionism scale.*p < 0.05; **p < 0.01; ***p < 0.001.

The OCI-R Ordering was significantly, moderately, and positively correlated with PROCSI Social Abilities (*r* = 0.35, *p* < 0.001) and PROCSI Emotional Stability was significantly, moderately, and positively correlated with MPS Preoccupation for errors and doubts about actions (*r* = 0.35, *p* < 0.001; see Table 5 for more detailed results).

A statistically significant moderate correlation emerged between MPS Preoccupation for errors and doubt about actions and PROCSI Emotional Stability (*r* = 0.35, *p* < 0.001; Table 4).

Statistically significant strong positive correlations emerged between PID-5 Detachment and all the ROCI subscales (Table 5). Moderate statistically significant positive correlations emerged for PID-5 Negative Affect, PID-5 Disinhibition, and all the ROCI subscales. See Table 5 for more detailed results.

Table 5 shows the moderate statistically significant positive correlations emerged between OCI-R Obsessing and ROCI Love for Partner (*r* = 0.46, *p* < 0.001), ROCI Adequacy of the Relationship (*r* = 0.50, *p* < 0.001), ROCI Love of Partner (*r* = 0.39, *p* < 0.001), and ROCI Total (*r* = 0.49, *p* < 0.001).

A weak statistically significant negative correlation emerged between MPS High Motivational Standards and ROCI Adequacy of the Relationship (*r* = −0.23, *p* < 0.05; Table 5).

### 3.5 Multiple linear regression with PROCSI scores as dependent variable

A multiple linear regression with PROCSI scores as the dependent variable and the scores on the PID-5, OCI-R Total, and MPS as predictors was conducted in the heterosexual group. As shown in Table 6, scores on PID-5 Antagonism (*B* = 0.41, *t* = 5.14, *p* < 0.001) and PID-5 Psychoticism (*B* = 0.28, *t* = 3.57, *p* < 0.01), and scores on MPS Preoccupation for errors and doubts about actions (*B* = 0.32, *t* = 4.11, *p* < 0.001) are statistically significant predictors of PROCSI scores. PID-5 Antagonism (*B* = 0.40, *t* = 4.73, *p* < 0.001), PID-5 Detachment (*B* = 0.28, *t* = 3.24, *p* < 0.01), and OCI-R Total (*B* = 0.19, *t* = 2.23, *p* < 0.05), as shown in Table 6, are statistically significant predictors of PROCSI scores in the LGB group.

**Table 6:** Multiple linear regression with PROCSI as the dependent variable (*n* = 200).

	***β***	***t***	***p***
**Heterosexual group (*n* = 98)**			
PID-5 antagonism	0.41	5.14	<0.001
MPS concern over mistakes and doubts about actions	0.32	4.11	<0.001
PID-5 psychoticism	0.28	3.57	<0.01
MPS parental expectations and criticism	0.13	1.31	0.20
PID-5 disinhibition	0.13	1.04	0.30
PID-5 detachment	0.09	0.93	0.36
PID-5 negative affect	0.06	0.74	0.46
OCI-R total	0.03	0.31	0.76
MPS personal standards	−0.01	−0.12	0.90
**LGB group (*n* = 102)**			
PID-5 antagonism	0.40	4.73	<0.001
PID-5 detachment	0.28	3.24	<0.01
OCI-R total	0.19	2.23	<0.05
MPS organizations	0.12	1.33	0.19
PID-5 disinhibition	0.07	0.79	0.43
MPS personal standards	−0.06	−0.63	0.53
MPS parental expectations and criticism	0.04	0.48	0.63
PID-5 psychoticism	0.04	0.46	0.65
PID-5 negative affect	−0.04	−0.35	0.73
MPS concern over mistakes and doubts about actions	0.00	0.00	0.99

PID-5, personality inventory for DSM-5; PROCSI, partner-related obsessive-compulsive symptom inventory; OCI-R, obsessive compulsive inventory – revised; MPS, multidimensional perfectionism scale. Homosexual group: *R*^2^ = 0.47; *R*^2^_corrected_ = 0.46; ESS = 11.00 LGB group: *R*^2^ = 0.33; *R*^2^_corrected_ = 0.31; ESS = 9.74.

### 3.6 Multiple linear regression with ROCI scores as dependent variable

A multiple linear regression with ROCI scores as the dependent variable and the scores on the PID-5, OCI-R Total, and MPS as predictors was conducted in the heterosexual group. As shown in Table 7 scores on PID-5 Antagonism (*B* = 0.25, *t* = 2.10, *p* < 0.05), PID-5 Disinhibition (*B* = 0.27, *t* = 2.31, *p* < 0.05), PID-5 Negative Affect (*B* = 0.28, *t* = 3.60, *p* < 0.01), and scores on MPS Preoccupation for errors and doubts about actions (*B* = 0.33, *t* = 3.80 *p* < 0.001) are statistically significant predictors of ROCI scores. In the LGB group PID-5 Detachment (*B* = 0.44, *t* = 5.18, *p* < 0.001), PID-5 Disinhibition (*B* = 0.26, *t* = 3.15, *p* < 0.01), and OCI-R Total (*B* = 0.17, *t* = 2.13, *p* < 0.05) were found to be statistically significant predictors of ROCI scores (Table 7).

**Table 7:** Multiple linear regression with ROCI as the dependent variable (*n* = 200).

	***β***	***t***	***p***
**Heterosexual group (*n* = 98)**			
MPS concern over mistakes and doubts about actions	0.33	3.80	<0.001
PID-5 negative affect	0.28	3.60	<0.001
PID-5 disinhibition	0.27	2.31	<0.01
PID-5 antagonism	0.25	2.10	0.20
MPS personal standards	−0.15	−1.50	0.14
MPS parental expectations and criticism	0.13	1.33	0.19
PID-5 detachment	0.03	0.31	0.76
PID-5 psychoticism	0.01	0.15	0.88
OCI-R total	0.01	0.11	0.91
**LGB group (*n* = 102)**			
PID-5 detachment	0.44	5.18	<0.001
PID-5 disinhibition	0.26	3.15	<0.01
OCI-R total	0.17	2.13	<0.05
PID-5 psychoticism	−0.17	−1.58	0.12
PID-5 negative affect	0.09	0.69	0.49

PID-5, personality inventory for DSM-5; PROCSI, partner-related obsessive-compulsive symptom inventory; OCI-R, obsessive compulsive inventory – revised; MPS, multidimensional perfectionism scale. Heterosexual group: *R*^2^ = 0.47; *R*^2^_corrected_ = 0.46; ESS = 11.00 LGB group: *R*^2^ = 0.41; *R*^2^_corrected_ = 0.39; ESS = 7.94.

## 4 Discussion

The present study aimed to investigate the differences regarding the presence of psychological characteristics referring to relationship-centered and partner-focused obsessive-compulsive traits, perfectionism, and personality traits between a group of heterosexual individuals and a group of LGB individuals and to explore the role of perfectionism and personality in these symptoms in both groups.

Our results show that LGB individuals report greater romantic relationship obsessive compulsive symptoms, specifically feelings of doubt regarding the partner’s love, greater episodes of negative emotions (Negative Affectivity) and antagonistic behavior (Antagonism), and greater perfectionism traits (Perfectionism) compared to heterosexual individuals.

These results seem to confirm our first hypothesis. Indeed, the LGB group reports greater romantic relationship obsessive compulsive symptoms; the obsessions regarding partner’s love seem to be most significant. This result could be explained expanding the hypothesis of Patterson and Riskind (2010) according to which cultural stigma toward the LGB community could influence the psychological health, social, and personal perception that the person has of the couple. This cultural stigma could lead LGB individuals to search for confirmation of their partner’s love to reassure themselves of their relationship. This behavior could explain the higher tendency of LGB individuals to report a greater tendency to focus on their partner’s love. Further, the presence of insecure attachment behaviors linked to dysfunctional cognitive processes related to OCD, could determine doubts regarding the partner (Lazarov et al., 2010). Indeed, fear of abandonment and difficulty in trusting others – characteristics of insecure attachment styles - could compromise functional coping strategies and increase intrusive thoughts (Doron et al., 2009, 2014). However, in discord with our hypothesis, no differences emerged regarding partner-related traits: therefore, doubts and compulsive behaviors regarding specific partner characteristics did not differ in our sample.

The presence of greater antagonistic traits in the LGB group is not in line with previous research, and is also in discord with our second hypothesis, that shows that transgender individuals tend to report less maladaptive personality traits (Anzani et al., 2020). However, the study in question took into consideration gender differences and used a structured clinical interview to diagnose a Personality Disorder, therefore, future studies could account for such variables.

The results confirm our third hypothesis: LGB individuals report greater maladaptive perfectionism, specifically greater concerns over mistakes and doubts about actions, greater Concern with Precision, Order and Organization and greater perceived parental expectations and criticism. This result is in line with previous research (Ying et al., 2022).

Correlation coefficients between the scores on the PROCSI, ROCI, MPS, and OCI-R seem to confirm our hypothesis that perfectionism may be linked with relationship obsessive compulsive symptoms. More specifically, perfectionism seems to be associated with greater partner-oriented obsessive-compulsive traits. This is in line with the fact that most of the perfectionistic dimensions significantly correlated with relationship-focused and partner-focused obsessions; more specifically, greater are the doubts regarding errors, greater is the tendency to worry about the relationship. Perfectionism, therefore, seems to be predictive and this result confirms those of Lazarov et al. (2010): if an individual presents excessive standards, it is more probable that when the real experience deviates from “next-to perfect” standards these individuals present greater doubt and preoccupations regarding the appropriateness of the partner and of the relationship – in terms of “right” or “wrong.”

Results from the multiple linear regression model, in discord with our hypothesis, show that antagonism and detachment are related to partner-oriented obsessive-compulsive traits in LGB individuals, after controlling for general obsessive compulsive symptoms. Further, Detachment and Disinhibition personality traits are associated with relationship-oriented obsessive-compulsive traits in LGB individuals, after controlling for general obsessive-compulsive symptoms. According to the available literature (e.g., Russell et al., 2017), LGB individuals report greater prevalence rates of Borderline Personality Disorder (BPD) than heterosexual individuals. BPD is characterized by the presence of Antagonism and/or Disinhibition and Negative Affect (American Psychiatric Association, 2013). This data could explain our results: maladaptive personality traits, specifically antagonism, detachment, and disinhibition are related to relationship-oriented and partner-focused traits in LGB individuals. Disinhibition refers to the ability to self-regulate or self-control behavior (Clark and Watson, 2008), including emotion regulation strategies (Tice and Bratslavsky, 2000) that successfully regulate emotions or determine problematic emotional reactions (Gratz and Roemer, 2004). Emotion regulation guides the course of interpersonal relationships (Frijda and Mesquita, 1994), and in sexual minority couples these strategies could be influenced by stigma-related stressors (Hatzenbuehler et al., 2009). A greater prevalence of social isolation, a characteristic of Detachment (American Psychiatric Association, 2013), in sexual minorities has been found by different studies (Gilman et al., 2001; Safren and Pantalone, 2006; Potoczniak et al., 2007). Regarding Antagonism, as previously mentioned, this result is in discord with previous research. However, ours is a community sample, therefore we did not take into consideration the presence of a specific psychiatric diagnosis, rather the presence of specific personality or relationship obsessive-compulsive traits.

Contrary to our hypothesis, perfectionism is not associated with relationship-focused or partner-oriented obsessive compulsive traits in the LGB community. This result is not in line with previous research that has found that perfectionism was associated with ROCD traits (Doron et al., 2012a,b, 2016). Specifically, Doron et al. (2012a,b) found moderate correlations between perfectionism and relationship-oriented/partner-focused obsessive-compulsive symptoms, and found a small-moderate correlation between relationship-oriented/partner-focused ROCD symptoms. However, these results were obtained on community samples and not on a sexual minority group. These discrepancies in the sample characteristics could explain the divergence in the results.

Finally, results from the multiple linear regression model show that Antagonism, Psychoticism, and concerns about mistakes and doubts about actions are related to partner-oriented obsessive-compulsive traits in heterosexual individuals; while concerns over mistakes and doubts about actions, negative affect, and disinhibition are related to relationship-focused obsessive-compulsive traits in heterosexual individuals. In fact, narcissistic personality traits – characterized by the presence of Antagonism in terms of grandiosity and attention-seeking (American Psychiatric Association, 2013) – have shown to increase doubts regarding partner’s characteristics (i.e., morality, physical appearance, social skills, emotional stability, competence, and intelligence), possibly due to the tendency to assign excessive, non-obtainable personal standards to one’s partner (Tinella et al., 2023). Relationship-focused obsessive-compulsive traits are characterized by the presence of perfectionistic worries and erroneous beliefs about being in the wrong relationship or being alone (Melli et al., 2018a).

## 5 Limits

First, our sample size is relatively small and does not include a clinical group, this is a very important limitation. Therefore, the results here obtained are not generalizable to patients with OCD because the participants derive from the general population. It is also worth noting that we did not take into consideration psychological variables that are potentially associated with the minority status of LGB or sexual minority individuals (i.e., interiorized homophobia, acceptance; see – Meyer, 2003; Rostosky and Riggle, 2017).

Second, our study adopts a cross-sectional design, therefore, all the limits associated with this design are implied. Third, within the LGB group we did not compare differences regarding symptomatology between homosexual and bisexual individuals, and we did not consider gender differences. Fourth, we did not take into consideration specific facets of personality given that our relatively small sample would not have been adequate to evaluate a larger number of variables. Finally, we did not use a semi-structured clinical interview to verify the presence of OCD, ROCD or any other psychopathological diagnosis in the sample. The use of self-report measures does not permit us to determine the presence or absence of a specific diagnosis.

## 6 Conclusion

This is the first study to investigate ROCD in the LGB community. Our results highlight that amongst the relationship- and partner-focused obsessive-compulsive symptoms, only feelings of doubt regarding the partner’s love were higher in the LGB group as compared with the heterosexual one. Specific personality traits were higher amongst LGB as compared with the heterosexual group, i.e., Negative Affect, Antagonism and perfectionism. In the LGB group, romantic relationship obsessive compulsive symptoms were related to Detachment and Disinhibition personality traits. Adopting a dimensional approach that considers romantic relationship symptoms and personality traits as dimensional constructs, rather than categorical ones, implies not the presence of stable traits that define a disorder, but possible ways of functioning that are potentially subject to change. This might be particularly important when considering prevention interventions and public health policies, where sexual minorities are involved.

## Data availability statement

The raw data supporting the conclusions of this article will be made available by the authors, without undue reservation.

## Ethics statement

The studies involving humans were approved by Siena Hospital Ethics Committee. The studies were conducted in accordance with the local legislation and institutional requirements. The participants provided their written informed consent to participate in this study.

## Author contributions

AP was responsible for final editing and project coordination. All authors contributed to the article and approved the submitted version.
